# EHealth and Its Role in Supporting Audiological Rehabilitation: Patient Perspectives on Barriers and Facilitators of Using a Personal Hearing Support System With Mobile Application as Part of the EVOTION Study

**DOI:** 10.3389/fpubh.2021.669727

**Published:** 2022-01-14

**Authors:** Louisa Murdin, Mark Sladen, Hannah Williams, Doris-Eva Bamiou, Athanasios Bibas, Dimitris Kikidis, Apostolis Oiknonomou, Ioannis Kouris, Dimitris Koutsouris, Niels H. Pontoppidan

**Affiliations:** ^1^Guy's and St Thomas' NHS Foundation Trust, London, United Kingdom; ^2^University College London, UCL Ear Insitute and UCLH Biomedical Research Centre, National Institute for Health Research, London, United Kingdom; ^3^Department of Otorhinolaryngology - Head & Neck Surgery, National and Kapodistrian University of Athens, Athens, Greece; ^4^Athens Medical Centre, Athens, Greece; ^5^National Technical University of Athens, Athens, Greece; ^6^Eriksholm Research Centre, Snekkersten, Denmark

**Keywords:** eHealth, teleaudiology, hearing loss, hearing aids, mobile phone application, public health

## Abstract

**Background:**

Hearing loss is a major public health challenge. Audiology services need to utilise a range of rehabilitative services and maximise innovative practice afforded by technology to actively promote personalized, participatory, preventative and predictive care if they are to cope with the social and economic burden placed on the population by the rapidly rising prevalence of hearing loss. Digital interventions and teleaudiology could be a key part of providing high quality, cost-effective, patient-centred management. There is currently very limited evidence that assesses the hearing impaired patient perspective on the acceptance and usability of this type of technology.

**Aim:**

This study aims to identify patient perceptions of the use of a hearing support system including a mobile smartphone app when used with Bluetooth-connected hearing aids across the everyday life of users, as part of the EVOTION project.

**Methods:**

We applied a questionnaire to 564 participants in three countries across Europe and analysed the following topics: connectivity, hearing aid controls, instructional videos, audiological tests and auditory training.

**Key Findings:**

Older users were just as satisfied as younger users when operating this type of technology. Technical problems such as Bluetooth connectivity need to be minimised as this issue is highly critical for user satisfaction, engagement and uptake. A system that promotes user-controllability of hearing aids that is more accessible and easier to use is highly valued. Participants are happy to utilise monitoring tests and auditory training on a mobile phone out of the clinic but in order to have value the test battery needs to be relevant and tailored to each user, easy to understand and use. Such functions can elicit a negative as well as positive experience for each user.

**Conclusion:**

Older and younger adults can utilise an eHealth mobile app to complement their rehabilitation and health care. If the technology works well, is tailored to the individual and in-depth personalised guidance and support is provided, it could assist maximisation of hearing aid uptake, promotion of self-management and improving outcomes.

## Introduction

Within the hearing health services, finding ways to support a high quality modern healthcare framework using innovations afforded by the internet and smartphones is central to dealing with ever-rising demands (1–6).

The global increase in prevalence of hearing loss is a key factor in this evolution. Hearing loss is increasingly identified as a public health concern. It is estimated that the number of people with disabling hearing loss (defined as hearing loss >40dB in the better hearing ear for adults and >30dB in the better hearing ear for children) worldwide stands at 466 million and by 2050 is predicted to rise to 900 million (7). Although hearing loss prevalence rises with increasing age, the figure includes 1.1 billion people aged 12–35 years who are at risk of hearing loss due to excessive noise exposure (7).

This increase in the prevalence of hearing loss will place a further social and economic burden on the hearing impaired population particularly and society in general (7–10). Globally there is a shortage of adequately trained audiologists to deal with this increasing demand (11).

The most common intervention to tackle the impact of hearing loss for an individual is a hearing aid and the benefits are strongly evidence based (12). However the uptake of such devices has been very low and, of those people fitted, up to 40% do not use them or fail to gain optimal benefit from them (13).

The reach of the internet means that people with hearing loss and their families and carers are able to access a vast amount of information and so have the potential for greater awareness and expectations of healthcare (14, 15). Furthermore there is demand for healthcare that supports the 4 “P”s: *personalised* on-demand service, *predictive* service through analytics, *preventative* service through monitoring, and *participatory* service to empower and reduce costs (16). This is in order to facilitate patient-centred care and promote self-efficacy.

Currently audiology does not adhere well to the 4 “P”s model (17). In the light of this, audiology care needs to re-evaluate the role it plays in the amelioration of hearing impairment, utilising a range of auditory rehabilitation services, rather than simply administering diagnostic testing and hearing aid dispensing, for example, by offering key skills and support such as psychological counseling, auditory training and promoting self-management (18, 19).

What, then, is the solution to the problem of increasing demand, low uptake of effective interventions and higher expectations of care? Can the issue be addressed by the use of digital health solutions? Use of mobile phone applications within healthcare is rising (10) and the number of audiology applications in particular is abundant (20). It has been found that increasing numbers of people with a hearing impairment are using the internet and there is an increasing popularity of personal smartphone usage (12, 21, 22).

One aspect of change in access and delivery of modern healthcare is the use of eHealth or tele-health, known in the hearing care domain as tele-audiology. The World Health Organisation describes eHealth as “*the use of electronic means to deliver information, resources and services related to health. It covers many domains, including electronic health records, mobile health, and health analytics, amongst others. eHealth can put information in the right place at the right time, providing more services to a wider population and in a personalised manner*” (23). Tele-audiology can be used to support services such as data collection; imparting education, training and information to hearing healthcare professionals, adults with hearing disorders and parents of children with hearing impairment; screening for hearing loss and auditory disorders; diagnostic testing; auditory rehabilitation including counseling, auditory training and hearing aid support (10, 24, 25). Teleaudiology could in theory also help improve access to hearing services for a wider population. This is especially important for people living in remote locations but also to helps patient groups at high risk of becoming lost to follow-up e.g. for socioeconomic reasons, accessibility, unsuccessful treatment outcomes (8, 26–28).

Tele-audiology, can improve the flexibility, efficiency, cost-effectiveness and quality of audiology care (10, 29–31), which can help extend audiological services and aspire to deliver care according to the “anytime, anywhere” principle (32, 33), Additionally tele-audiology facilitates the application of personalised audiological rehabilitation, supports the advancement of public health policies, engages patients increasing their motivation and promotes behaviour change (34). A recent systematic review concluded that further research is needed to investigate the benefits of individual elements of tele-audiology rehabilitative services (35).

However, relatively few studies have investigated the patient or user perspective of eHealth applications in audiology (8). Personalisation and control of hearing aid settings was very important to participants in one study (36). Another study has found that patient experience and satisfaction with a hybrid audiology service using both face to face and remote elements were equally high and positive in both online and face-to-face service offerings (37). A study as long ago as 2005 surveyed 116 audiology patients and showed the majority of them had not heard of eHealth, although this may well have changed increased in succeeding years (38). Furthermore smartphone applications are often designed in relation to technological constraints and not primarily to user needs (39). Patient involvement with eHealth is highly linked with previous knowledge and awareness and so user experiences should be integrated by design to develop effective patient education (38). There is an emotional and psychological dimension to healthcare experience (40) and the impact of user emotion upon using these eHealth tools is often neglected, but it is a very important consideration for achieving high uptake and utilization (41). For example, if the technology does not work reliably the user is unlikely to fully engage with it if at all and therefore it will not be adopted or used (42). A poor experience with one e-Health system could also have negative consequences for uptake of different systems.

The EVOTION project is an international, multicentre cross-disciplinary complex multi-stranded project targeted at improving public health decision making for hearing care (43). One arm of EVOTION involved collection of real-time data through specialised hearing aids and an associated mobile phone app connected via Bluetooth. The EVOTION app also provided participants with self-administered testing, auditory training and noise exposure reporting, hearing aid control and informational material both for participant individual clinical benefit and for data collection purposes.

This paper reports the user perspectives on experience with this particular group of technologies and applications, and to identify some of the key barriers and facilitators to the successful adoption of this type of eHealth intervention within audiology clinical settings across Europe.

## Materials and Methods

### Study Population

1065 participants for the EVOTION study were recruited from six audiology centres across three countries in Europe, namely Greece, the United Kingdom and Denmark (Table 1). The centres were providing healthcare within different models. All participants were provided with EVOTION hearing aids, which were Oticon OPN devices modified for the purposes of data collection, and a smartphone pre-installed with a set of applications that connected to the hearing aids. This system collected data on variables including hearing aid usage and sound environment which is not described in this paper. Participants had to use the application on the phone provided and not on their personal device for ethical (data governance) and technical reasons. The phone could be used for other purposes if the participant wished.

**Table 1:** Participant recruitment at different institutions.

**Name of institution**	**Guy's and St Thomas' NHS Foundation Trust (GST)**	**University College London (UCL)**	**University of Athens (UOA)**	**Athens Medical Centre (AMC)**	**James Paget University Hospitals NHS Foundation Trust (JP)**	**Oticon (OTC)**
Country	UK	UK	Greece	Greece	UK	Denmark
Access model	Via National Health Service (NHS England).	Via National Health Service (NHS England).	Via University Clinic	Private	Via National Health Service (NHS England).	Research centre; subsidised care
Number of eligible EVOTION participants for this study	383	87	308	209	47	31
Number of participants completing feedback study (% eligible)	294 (77%)	16 (18%)	178 (58%)	51 (24%)	16 (47%)	9 (29%)
Age range in years; mean	18–87; 60	46–80; 65	18–95; 66	20–86; 60	46–82; 60	59–81; 72
Gender F:M	51:49	56:54	46:54	42:58	41:59	13:87

EVOTION participants were required to be over the age of 18 and have a basic understanding of oral and written English, Danish or Greek as relevant to the recruiting country. Exclusion criteria included cognitive impairment [Montreal Cognitive Assessment (44), also known as MoCA, score less than 22] and visual impairment severe enough to preclude use of the visual display on the app. Eligibility was determined by the presence of unilateral and/or bilateral mild-to-profound sensorineural hearing loss, or a mixed hearing loss and willingness to wear the EVOTION hearing aid/s for at least two hours a day for the duration of the study. Participants were not excluded on the basis of prior hearing aid or smartphone experience. There are defined fitting criteria for EVOTION hearing aids which related to audiometric eligibility criteria, because the hearing aids used in the study were a receiver in-the-ear type model with a maximum output of 85dB using Oticon minifit domes. The devices were fitted by trained audiologists and participants had as much fine tuning support as they required. Inclusion within the study was dependent on these hearing aids being an applicable fit. Participants were identified as they were attending one of the participating Audiology clinics (Table 1).

The study was reviewed and approved by the Health Research Authority (HRA-UK), received ethical approval from the Research Ethics Committee (National Health Service NHS REC led in UK) and additional local institutional approvals accordingly.

### Mobile Application Tests and Activities

**Figure 1:**
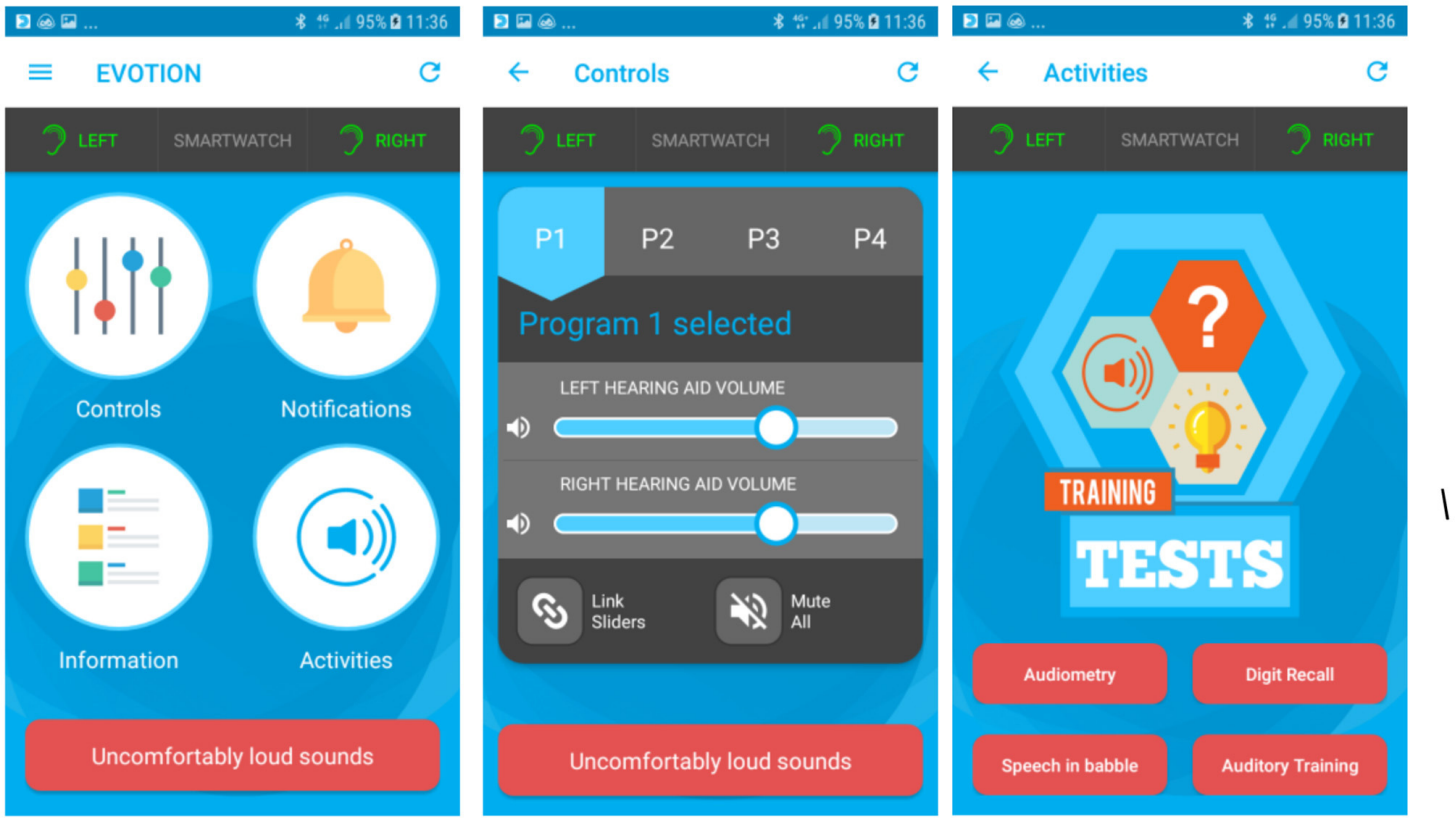
EVOTION mobile app screens: Left panel shows all available functions. Middle panel shows the hearing aid controls. Right panel shows the four available tests and activities.

There were several different types of tests and activities on the Evotion system. Some of these were designed primarily as data collection tools for the EVOTION study and others were more clearly tools that participants could benefit from directly. The self-administered tests within the EVOTION app aimed to assess factors important for monitoring hearing and listening including cognitive abilities. They included aided pure-tone 4 kHz audiometry, speech-in-babble and digit recall as well as an auditory training module (43). Speech perception could be monitored using the self-administered tests. The hearing aid controls allowed the user to remotely change the volume and program setting using the mobile phone interface. Finally, informational material was provided in the form of short hearing aid instructional videos to educate and actively engage the user. The user interface of the system is shown in Figure 1.

### Controls

In the EVOTION system the user had the ability to change the settings on both hearing aids simultaneously (one hearing aid only if a unilateral fit). The settings consisted of four listening programs that could be selected by pressing the corresponding button. The programs could be used based on participant preference within different listening environments. The user could also change the volume of the hearing aids up and down using a slider button.

### Instructional Videos

The user could access videos showing how to perform hearing aid maintenance tasks, for example changing the minifit dome or receiver wax guard.

### Audiometry

This test measured pure tone threshold at 4 kHz. Temporary threshold shifts at 4 kHz are indicative of exposure to potentially hazardous loud sounds. The app used a form of sweep audiometry, where the intensity of the tone was varied continuously. If the user could hear the tone, they press the on-screen button and then release when they can no longer hear it. The logged sound environment data then enabled the mobile to calculate the actual level of the tone at the EVOTION HAs and thus calibrate the threshold. The participants were asked to complete this when they felt they were exposed to potentially hazardous loud sounds. Users received instant feedback on the results of the test. The trigger for this was a detected sound level high enough to cause a temporary or permnanet hearing change according to published data (45).

The self-measured 4 kHz PTA was an aided threshold: a probe signal (the tone) was emitted from the EVOTION mobile phone's loudspeaker and then travelled through the air to the EVOTION Hearing Aid, where the level was measured, and the signal was amplified according the patient's audiogram. The EVOTION Mobile phone controlled the intensity (SPL) of the probe tone and recorded patient response as to when the tone was audible and when it was not.

The patient was instructed to press the on-screen button when s/he could hear the tone and release the button again when s/he could not hear the tone anymore. They were instructed to place the mobile phone on the table in front of them and to wear both hearing aids during the test. The patient was instructed to keep positions of the phone, the patient and hearing aids constant during the test. During the test the app changed the hearing aid program to the one in which the least amount of processing was applied. At the end of the test the original settings the patient had been using were restored.

The mobile app recorded the patient's response and sent the results to the EVOTION server. When the test ended, the mobile app restored the volume settings to those before the audiogram measurements.

**Figure 2:**
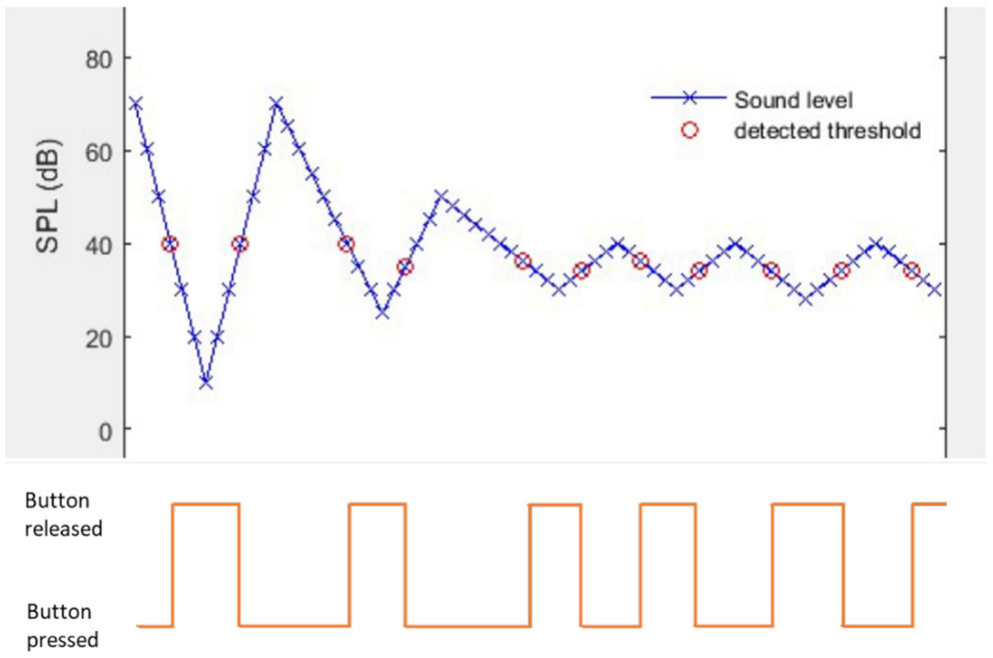
Bekesy tracing and patient response to EVOTION self administered 4kHz pure tone audiometry.

The test followed the Békésy measurement paradigm where the probe signal was a 4 kHz tone with time-varying level controlled by the EVOTION mobile phone. Figure 2 gives an example of one trial where the threshold was just below 40 dB SPL. Initially the tone started at an audible level, here 70 dB SPL, then it drops in steps of 10 dB. As it got below 40 dB the patient indicated that it was not audible (red circle), and the mobile phone started to increase the level in 10 dB steps from 2 steps below the inaudible tone, 10 dB. As the level reached 40 dB again the patient responded that the tone was audible. From there on the step size is halved to 5 dB (later halved once more to 2.5 dB).

When $\hat{N}$ detection thresholds had been recorded the threshold was calculated as follows


$$\begin{array}{l} Threshold_{\hat{N}}=mean\left(detection\;thresholds\right)\\ \;=\;\frac{1}{\hat{N}}\overset{\hat{N}}{\underset{i=1}{\sum }}detection\;threshold_{i}\\ SD_{\hat{N}}=\sqrt{\frac{1}{\hat{N}}\overset{\hat{N}}{\underset{i=1}{\sum }}{\left(detection\;threshold_{i}-Threshold_{\hat{N}}\right)}^{2}} \end{array}$$

Detection thresholds further away from Threshold 1 than $2\times SD_{\hat{N}}$ were considered outliers and discarded, so the final Thresholdwas calculated from the N remaining detection thresholds.


$$\begin{array}{l} Threshold=\frac{1}{N}\overset{N}{\underset{i=1}{\sum }}detection\;threshold_{i} \end{array}$$

### Digit Recall

This test assessed working memory and cognitive ability. Participants were asked to listen to a sequence of numbers and then type it into the keypad, starting with a two-digit figure and gradually increasing the number of digits. The participants were asked to do the Forward test at least twice and the Backward test twice, once in the first week and once four weeks later.

The digit recall test that was implemented in EVOTION was based on the digit span subtest of the Wechsler Adult Intelligence Scale (WAIS) IV (46). Digits from 1 to 9 were recorded by a male native English speaker. Pairs of digit sequences were played at a comfortable and the user had to type in the sequences in the correct order. There were 2 versions of the test, a forward and a backward version, where the listener has to type in the digits in the right or reverse order, respectively. On successful recall of at least one of the 2 sequences from each pair, the sequence increases by one digit (maximum 8 digits for forward and 7 for backward recall). Discontinuation occurs (i.e. the test ends) when both sequences are recalled incorrectly (i.e. at least one digit is incorrect). Equivalent digits 1 to 9 were also recorded in Greek and were implemented for the Greek version of the test with the exact same design as above.

### Speech-in-Babble

This test was developed based on an existing Speech-in-Babble test (47, 48) that employs eight lists of monosyllabic phonemically balanced meaningful English words and bisyllabic Greek words (due to lack of adequate number of monosyllabic words in the Greek language) as the speech stimulus presented with multi-talker babble as the masker. The participants were asked to complete this at least once in the first week and again four weeks later. The Danish participants were very few in number and were able to complete the test in English as a second language.

The words in noise test is largely based on the Speech-in-Babble (SiB) test that employs 8 lists of monosyllabic phonemically balanced meaningful English words as the speech stimulus presented with multi-talker babble as the masker. Each list contains 20- 25 words. These are spoken by a female native Southern-English speaker. Each word is delivered with 500 milliseconds of babble masker at the beginning and the end of the word itself (47).

Participants were instructed to place the mobile phone on a table in front of the patient and to wear both hearing aids during the test. The listener was asked to type in the word they heard. The Signal to Noise ratio (i.e. level of the target words vs. noise) is fixed throughout the test at 10 dB and performance was measured with the % correct responses. The English words that were used were those of the SiB test. During the test the app changed the hearing aid progam to the one in which the least amount of processing was applied. At the end of the test the original settings the patient had been using were restored.

Equivalent material was recorded in Greek specifically for the purposes of the project based on published Greek phonemically balanced word lists (49). Words in these lists are phonemically balanced, which means that they contain percentage of phonemes similar to the one recorded in the Greek language with analysis of a big sample of raw speech from various TV and radio shows. The associated speech audiometry test is used in every day clinical practice in Greek hospitals.

### Auditory Training

This was developed based on the Storey in Noise, an existing auditory training program using words in phrases spoken by adult female and male talkers (50). Phrases were taken from a connected narrative taken from books aimed at foreign learners of English with a background of continuous steady-state speech-shaped noise. The listener was asked to click on 1–3 keyword(s) present in the target phrase from a set of 2–6 options, each incorrect answer being phonetically similar to the target. Corrective feedback was given. The phrase was replayed every time a wrong choice is made. Every auditory training session lasted 15 min. Participants were asked to complete this at least three times a week for the first five weeks of participation.

The auditory training program in EVOTION was based on the Storey in Noise, that uses words in phrases from connected narratives spoken by adult talkers and presented in background noise (50).

Two texts were implemented in English: “Money for Sale” by M Hardcastle, and “Snowball”, with texts taken from books aimed at foreign learners of English. Three texts were implemented in Greek: Crazy Antonis (T$\rho \varepsilon \lambda \alpha \nu \tau \overset{´}{\omega }$ν*ης*, Π. $\Delta \overset{´}{\varepsilon }$λ*τα*), For Whom the Bell Tolls (E. Hemingway) and Perfume (Patrick Süskind). Each text was divided into phrases of 2–10 words. The number of phrases per text ranged from 300 to 2641. The median phrase length for each text was five words. For each phrase, between one and four potential target words were selected. Target words were primarily content words, although function words were used in a small proportion of phrases. Similar sounding foil words were chosen for each target that shared at least two phonemes with the target and were chosen so as to be plausible in the context of the narrative. Phrases were presented with a background of multi-talker babble noise. The participant listened to consecutively presented phrases and after each phrase saw a display containing keywords along with a number of alternatives/foils. Each phrase had up to 4 possible target words. The training ran for a 30 min session, subdivided into four blocks of 7.5 mins. The noise level adapted according to the errors made over the preceding 10 phrases. The initial SNR was set to 10 dB (i.e. target sound 10 dB higher than the noise). If the proportion of possible errors made was > 0.15 then the SNR for the next 10 phrases was increased by 3 dB, otherwise it was reduced by 3 dB.

### Feedback Questionnaire

A small sample of participants in one of the UK centres were interviewed to provide a preliminary outline of potential areas of concern arising from the use of the mobile application, and these were used to generate topics which in turn were developed into items on a questionnaire to assess participant perspective. The topics were: satisfaction with connectivity between the hearing-aid and the EVOTION app; use of hearing-aid controls on the app; instructional videos; and app auditory activities.

The final questionnaire asked participants to rate their satisfaction on a five-point Likert scale with seven different aspects of the EVOTION mobile-phone app: Bluetooth connectivity, hearing-aid controls, hearing-aid instructional videos, audiometry tests, digit recall tests, speech-in-babble tests and auditory training tests. A further question asked for their overall satisfaction with using the mobile phone app. Each question allowed for comments and an additional question at the end invited a free text response to outline any further comments or issues encountered.

This questionnaire was administered to participants at the end of the intervention period which was 12 weeks after being given the EVOTION interventions.

Participants were free to decide whether or not they wished to complete the feedback forms or to write in the comments sections.

The text comments from the EVOTION feedback forms were analysed with a thematic analysis method (51), using an inductive approach to identify any possible emergent themes about the efficacy and user-friendliness of the EVOTION mobile app technology (52). All comments were reviewed and assigned a code as they appeared in the data. Data was then reviewed on a line by line basis and initial codes refined to develop a coding framework agreed by the author subgroup (LM, HW, MS). All free text was then re-coded according to the refined coding framework. These were then used to identify themes and subthemes which were checked against each other and to the original data set. Quotations were extracted to illustrate themes and subthemes. The coding team (LM, HW and MS) met during this analysis period to compare, discuss and refine initial and final codings, and were encouraged to challenge assumptions and interpretations during this process.

All statistical analyses were conducted using Microsoft Excel 2013 and SPSS version 26.

## Results

### Quantitative Results

A total of 1080 people participated in the study across all centres, 1037 (96%) participants completed their follow-up appointments as part of the EVOTION protocol. Of these, 564 (54%) filled in and returned feedback forms (see Table 1). The four frequency average (mean of thresholds at 0.5, 1, 2 and 4 kHz) had a mean of 43 (SD 17) dB (left ear) and of 42 (SD 17.2) dB (right ear). The age distribution of the respondents for the feedback study was similar to those of the study as a whole (mean 63 years, SD 15).

One way ANOVA indicated no significant difference between clinics in terms of participant age (F = 1.561, *p* = 0.17). Fisher's exact test indicated no significant difference between clinics in terms of gender distribution (*p* = 0.176).

**Figure 3:**
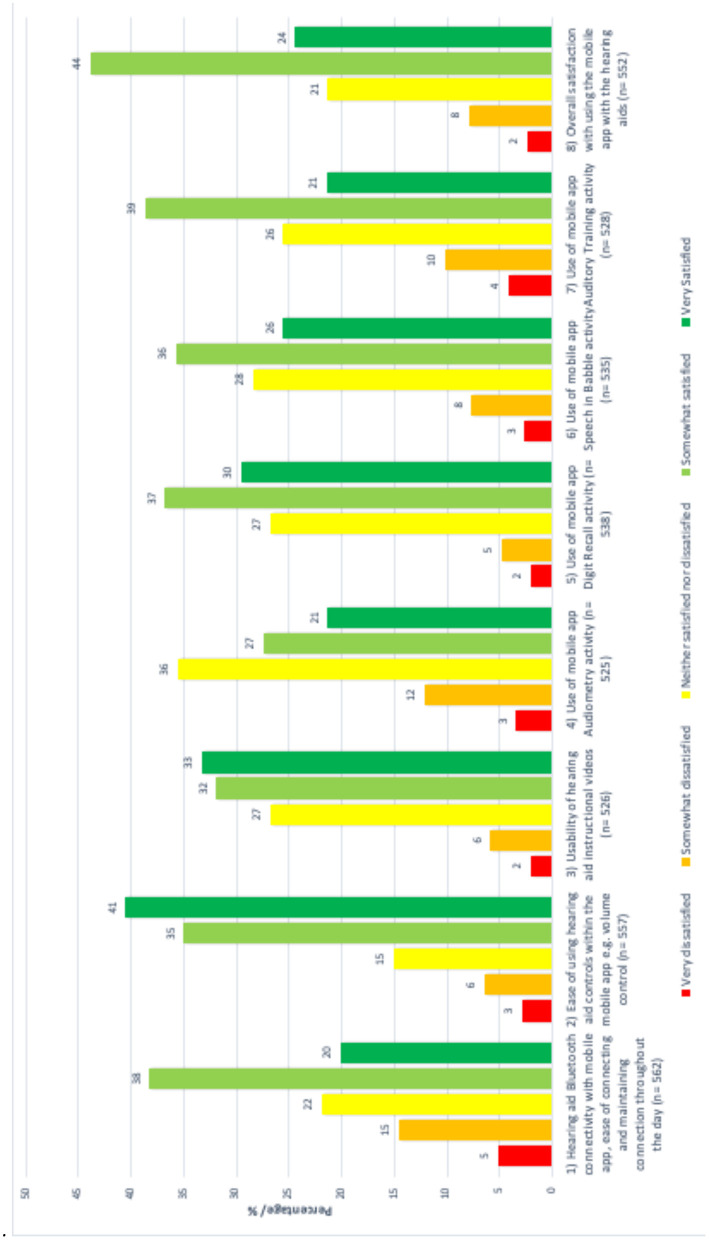
Distribution of feedback scores.

Not all participants submitted an answer for every question. Response completion and satisfaction levels are given in Table 2 and Figure 3.

**Table 2:** Response completion and satisfaction by feedback questionnaire item, according to country of origin and age (*n* = 564 returned questionnaires).

**Questionnaire item**	**Completed responses per item**	**Percentage completion %**	**% Satisfied (Overall)**	**UK % neutral/positive**	**Greece % neutral/ positive**	**Effect of country of origin (UK vs. Greece) *P* value; Chi squared test statistic**	**Effect of age *P* value; Chi squared test statistic**
Connectivity	562	99.6	58	87	95	<0.001*; 53.6	0.05*; 12.5
Controls	557	98.8	76	95	95	0.002*; 10.1	0.13; 10.0
Videos	526	93.3	65	95	89	0.01*; 6.6	0.16; 9.3
Audiometry	525	93.1	48	90	76	<0.001*; 18.2	0.70; 3.8
Digit recall	538	95.4	67	89	97	<0.001*; 20.1	0.38; 6.4
Speech-in-babble	535	94.9	62	89	91	0.397	0.74; 3.5
Auditory training	528	93.6	60	85	85	0.952; 0.004	0.07; 11.7
Other	552	97.9	x	x	x	x	0.24; 8.0
Free text comments	459	81.3	x	x	x	x	x

The asterisk (*) denotes significance at the 5% level. The symbol “x” denotes an invalid field, as free text comments and the category “other” were not suitable for all types of analysis.

The rate of respondents saying they were satisfied or very satisfied overall was 68%. There was no effect of gender on satisfaction for any item.

**Figure 4:**
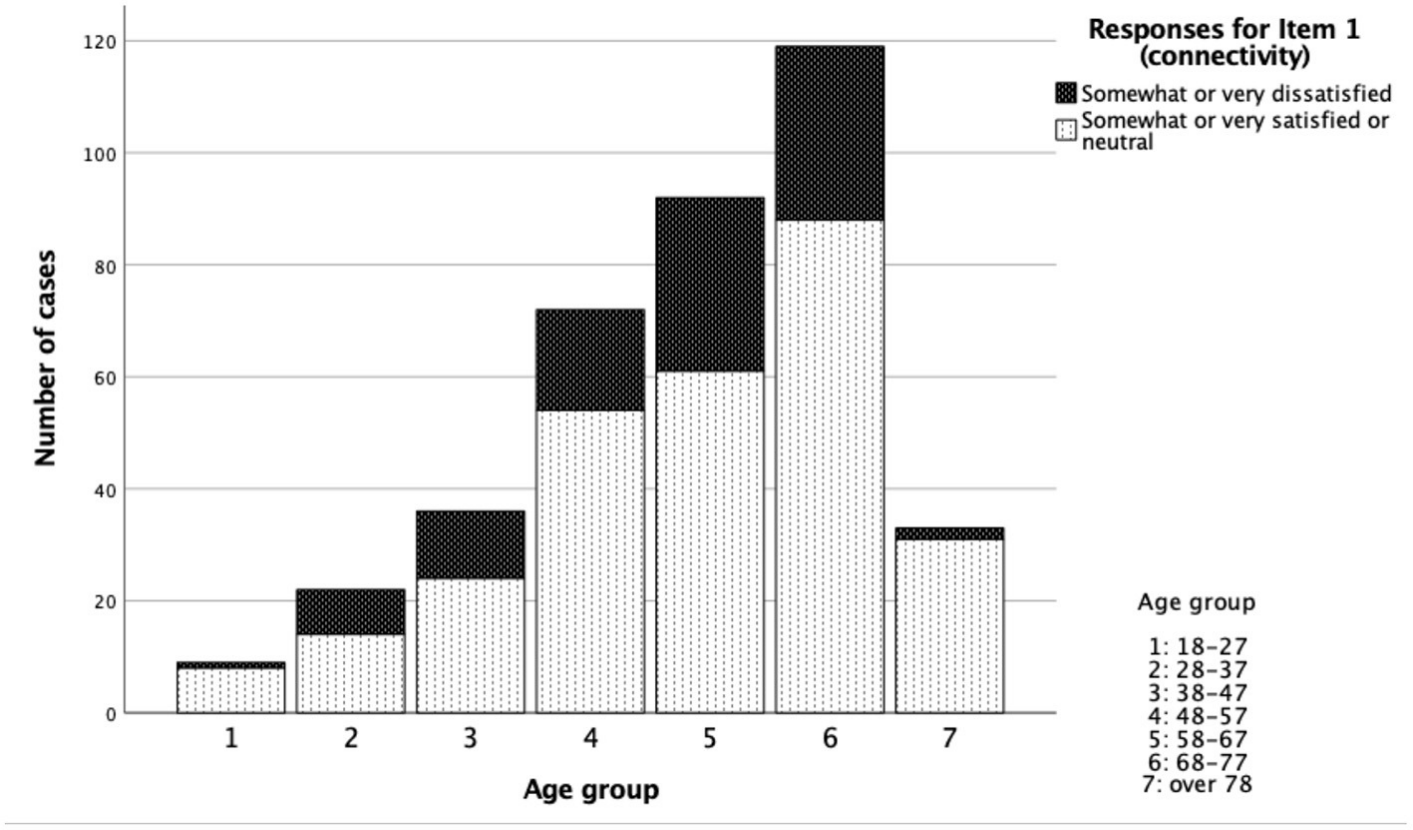
Satisfaction with Item 1 (connectivity) by age.

Age effects were analysed by grouping participants into age groups by decade, starting at 18 years which was the minimum age for participation. There was no significant difference in responses for each item across age groups, except for item 1 (connectivity), where there was a significant difference with the oldest and youngest groups reporting higher rates of satisfaction than intermediate age groups (*p* < 0.05, Chi squared, Table 2). Satisfaction data with respect to age are illustrated in Figure 4.

To examine the effect of country of origin, the UK and Greek populations were compared. The Danish group were excluded from this analysis due to relatively small numbers. Greek participants were significantly more likely than UK participants to report satisfied or neutral views of all items other than the speech in babble (item 6) and auditory training (item 7) components, where satisfaction rates were similar (see Table 2). The UK clinics did not differ from each other on any item for satisfaction rates. On most items, the Greek clinics were also not significantly different from each other, although the from one clinic in Greece (AMC) participants had higher rates of satisfaction for 3 items (videos, audiometry and speech in babble) on initial analysis but these differences disappeared with multiple hypothesis correction (Bonferroni applied) apart from item 4, audiometry.

### Qualitative Results

Feedback questionnaires were given at the follow-up appointment, and participants were under no obligation to provide comments. These were grouped under topic headings that corresponded to the questionnaire items in the quantitative study (listed in Table 2 above). Responses within each topic were coded and then grouped into themes within each topic.

The codes were then examined for emergent themes, which were validated by returning to the data repeatedly and reviewing both the codes and the themes.

The answers to the “overall comments” question were reassigned to fall within the topic and coding framework wherever possible, to allow for a distribution of the thematic codes. Any comments that could not be coded in this way were allocated as Other. There were 56 “other comments”, which were also analysed for further themes that may not have been included in the original questionnaires.

**Figure 5:**
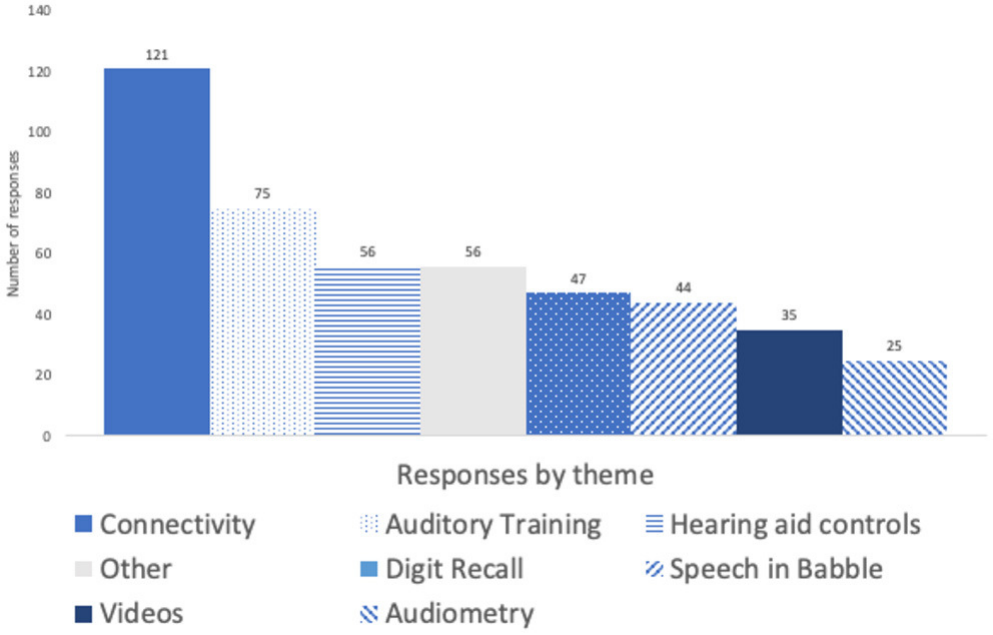
Free text comments volume (number of comments) grouped by topic.

Comment volume by topic is summarised in Figure 5 and Table 3 below.

**Table 3:** Example extract of comments from the data, giving an overview of topics and themes, are given below.

**Topic**	**Themes**	**Example comments**
1. Connectivity	Disconnects frequently	“The connection in the App dropped out at least once or twice a day” “Very erratic connections, despite trying various combinations of rebooting the app or hearing aids. Refresh buttons ineffectual”
	Frustration	“The connection with the app dropped out. It was irritating but easy to reconnect” “Doesn”t connect all that well and the phone has to be close by at all times. Annoying when you leave a room as it often doesn”t reconnect” “After being out of range, Bluetooth does not connect automatically – annoying!” “I keep losing connectivity which is starting to annoy”
	Disappointment	“Poor connection was disappointing”
2. Hearing Aid controls on app	Buttons froze/jumped	“Volume levels change after I have set them” “Sometimes doesn”t lock the “slider”, so end up with L/R out of sync” “Sometimes the volume control goes down” “Synching doesn”t seem completely reliable. One hearing aid volume seems to increase of its own accord” “Volume control fluctuates frequently” “Have found volume control alters under own volition”
	Useful	“Used this a lot, mostly to change between program 1 and 4” “Really easy – prefer this to using controls on hearing aids” “Really marvellous to be able to adjust volume etc so easily” “Much better than using controls on the hearing aid itself – too fiddly” “Excellent to be able to adjust volume settings on phone rather than fumble with aid”
	Did not use	“Didn”t want to carry phone. Used controls on HA” “I use the volume control completely via the earpiece as it is easier than using the mobile” “It would be good to change program without need of mobile app as if not connected can take a while when you want it to be immediate”
3. Instructional videos	Fine	“Clear and helpful”
	Not useful	“Didn”t find them useful”
4. Audiometry	Worked well	“Very easy to follow” “I enjoyed doing it, but fail to see how it is going to help me” “Very easy to follow” “This works very well”
	Did not work	“Frustrating when it refuses to work due to background noise” “Takes long to start” “It took a long time to get connected. It says 2 mins, but it still says 2 minutes after $\frac{1}{2}$ hour”
	Did not use	“Didn”t use – no uncomfortable noises” “I have not had a reason to use this. However, I note that it is extremely easy to inadvertently touch the “uncomfortably loud sounds” button which I have many times”
5. Digit Recall	Difficult	“Longer sequences present cognitive challenge” “Easy to use but 10 digits backwards is excessive!” “Always had difficulty in repeating numbers backwards” “Very difficult to put in long numbers backwards” “As the numbers increase, I find this challenging and in reverse order it is difficult.”
	Operational difficulties	“Started very quickly. Took me by surprise”
	Cannot see relevance of test	“The digit recall is rather weak, and I see little benefit in doing it when the other exercises appear to provide all of the things that it does, without such an unnecessary “concept” to hearing loss.” “Not sure how it”s relevant” “Longer sequences present cognitive challenge but no hearing issue”
6. Speech-in Babble	Difficult	“Takes a lot of concentration and a quiet area” “Background noise makes it difficult” “Quite difficult to hear with very loud background noise”
	Useful	“The most important of the four activities. Like real life” “I found the speech in babble frightening at first because it mimicked real-life situations where I couldn”t hear properly. I found it useful to acclimatise myself to this situation” “Makes aware of hearing challenges. Useful for others to hear too, to show what my hearing loss is like” “Used on day as instructed. Aware that my hearing with background noise is pretty limited”
	Cannot see relevance of test	“It felt a little long and took quite some time. The app doesn”t move on until the correct answer so it can be a lot of guesswork and many attempts” “I was getting high scores, e.g., 80%, mostly by guesswork. 10% would have been realistic”
7. Auditory Training	Storey crashes	“Storey stops halfway through, which is frustrating given time and effort” “Have experienced cut-off a few times and have to start again, which made the whole task twice as long” “On occasion got near the end of the task and the software threw me out – quite frustrating when busy” “Could do with a pause function. Froze on two occasions and had to go back to beginning”
	Too long/boring	“The sessions were way too long”
	Cannot see relevance of test	“Most answers can be obtained from context. Feel there is very little improvement” “Cannot understand 90% of it but guess answers according to the storey!” “Sometimes I find the words cannot be heard at all amongst the babble and I have to pick a word at random to hear them again”
8. Other	Practicalities	“Desperately need EVOTION app to work on my own phone”
	Battery life	“I found the batteries ran out very quickly”

### Bluetooth Connectivity

Bluetooth connectivity between the mobile app and the hearing aids was the most frequent free text subject for participants. There were 121 text comments. The vast majority were about problems incurred with the set-up/operation of the app. For some, it was a minor inconvenience; for others a source of frustration.

Some participants were able to re-establish the connection by switching their hearing aids off and on again (by opening the battery drawer). Others switched the mobile phone off and on again, and others re-paired the hearing aids with the mobile phone through the Bluetooth setting.

### Hearing Aid Controls

Participants in general liked controlling their hearing-aid volume and changing programs remotely, in some cases because the buttons on the hearing aids themselves require better dexterity but also because many people prefer not to draw attention to their hearing aids by touching them in public.

A minority of the comments (8% of those about the controls) indicated that people would rather control the hearing aids on the aids themselves.

Some people were not aware that they had the option of using the hearing aids to adjust the volumes/programs. For unilateral fittings the volume button was disabled because it could only go in one direction, whereas the app could be used to adjust the volume either way.

### Pure Tone Audiometry

This was the section of the questionnaire with the fewest and briefest answers. The calibration procedure was reported by some participants to be tricky because of the need for a quiet environment. Most comments discussed limited or non-use of this particular test or the encountering of technical issues completing the test.

### Instructional Videos

There were only 35 comments on the instructional videos for the hearing aids included with the mobile phone app. Half were positive, with the rest either non-specific negative comments or comments indicating they had not been used. Nobody criticised the content of the instructional videos. Participants were given clear verbal instructions and issued with printed instruction booklets at their hearing-aid fittings, so the need for instructional videos may have been minimal for some. The “how to change the wax guard” video was the most commonly used, reflecting a common need for an additional hearing-aid appointment.

### Digit Recall

A few people failed to see the relevance of digit recall, which put them off using it. People also found it was very difficult and memorising the digits in reverse order in particular was challenging for many. As with every aspect of the app, some of the comments reported on the activity freezing due to connectivity issues.

### Speech in Babble

The purpose of the Speech-in-babble test, an adaptive test that presents a word in background noise, was more apparent to most participants, with some of them commenting on how it related to their real-life difficulties with speech discrimination in noisy environments such as cafes and social groups.

A few participants felt that the scoring system was an inaccurate reflection of their ability because they believed they were having to guess. One suggested a progress bar would be a useful indicator to keep the participant motivated.

### Auditory Training

Some participants found the auditory training enjoyable but clear critical themes also emerged including the app crashing; the storey being too dull or long; some participants found it difficult and they felt they were guessing. Connectivity again had a big impact, particularly if the test crashed close to the end of the half-hour timeslot required to do the test, because the participant would have to start again which was disheartening.

Many participants questioned the validity of the test because their answers were guesses. However, this test is designed to be a contextual test, reflecting real-life listening environments in which the listener, particularly if hearing-impaired, has to fill in the sound they cannot hear by using contextual and acoustic cues they have heard.

Five themes were common to all the auditory activities and are summarised in Figure 5 below for comparison. The most common theme for Auditory Training was the test crashing whereas for Speech-in-Babble and Digit Recall it was the difficulty of the test content.

### Overall Satisfaction

When asked about their overall satisfaction with using the app with the hearing aids, the overarching message was that people liked having an app that they could use for controlling their hearing aids and they were well-disposed to doing the tests. The connectivity issues became an obstacle to this, however, and in some cases this inconvenience outweighed the benefit of having the device because it made it rather pointless carrying it around, since in the study the app was only available on the phone provided by the researchers and not on the participants' personal devices.

Participants wanted to do as well as possible and got frustrated when the tests did not work smoothly. They struggled more with the Auditory Training task than the Digit Recall and Speech in Babble tasks, not least because they were expected to spend far more time on it, in an environment with minimal background noise.

Other issues included frustration with the short battery life, which is a result of the phone connection draining the hearing-aid batteries; the frustration of carrying around two mobile phones; and problems for people with poorer eyesight with reading the possible answers on the auditory training tests.

Although the questionnaire did not specifically ask about the hearing aids, some of the most positive feedback was based on success with the hearing-aid technology.

## Discussion

Participants were positively satisfied across all items (except item 4-audiometry which was scored neutrally), with males and females being equally satisfied, and no difference in satisfaction across the different participating countries.

While there were no significant differences comparing age and scoring for most topics there was a statistically significant difference for item 1 (connectivity). This highlighted that the older participants were more satisfied with the connection between the smartphone and the hearing aids than most of the younger participants, except for the very youngest group. The youngest group were a small group relative to the others in the study because hearing loss which mainly affects older adults.

This finding around satisfaction with connectivity could indicate that the older group is more adept at dealing with technical issues. However, while previous work has suggested that older aged people are open and eager to adopt technology, they also lack confidence in their ability to use it. They are in general less aware of differences and similarities between different types of technology (53). It is also known that older users are more selective in the technology that they use. It may be that this group is less demanding or less expectant as they generally use a narrower range of technology and use it less frequently. However it is notable that the more frequent users of health care domain technology such as monitoring devices are older perhaps as this age group has a greater need for healthcare, or has more time available to spend learning to use new technologies than those of working age (54). Our results may be a reflection of this lack of experience and/or a greater need of the technology so subsequently any connectivity problems impacted the older users to a lesser degree.

There were fairly consistent differences in satisfaction level between Greek and UK participants with almost all elements, the Greek participants reporting higher levels of satisfaction than the UK participants other than for the audiometry function which the UK participants reported more positively. The reasons for this are not clear, but may have to do with different healthcare systems, different expectations of healthcare or other cultural factors. In any case this illustrates that acceptability of eHealth interventions is not uniform across different ethnic populations. Interestingly, the auditory training task was not rated differently between Greek and English participants, even though the test had components that could potentially be affected by native language.

Furthermore, the most common theme was about disappointment and frustration around connectivity. One user said in a very good summary of the overall picture, “I would strongly support the use of this, but for the fact that the connection keeps on failing”. This fits in with previous research that found ease of use and usefulness is important to users and is clearly associated with adoption and intention of use of technology (55, 56).

Controls were highly rated, with 76% of participants reporting satisfied or very satisfied, and there were a large number of positive comments, despite the negative influence of connectivity issues that impacted function. This observation is in keeping with previous studies (36). Participants found the volume control and program change via the smartphone very useful. It is known that even experienced users struggle to use the control buttons on the hearing-aid device itself as they can be awkward and small (57). Our study shows that a system that promotes user-controllability that is more accessible and easier to use is valued. These control changes are instantaneous and thereby reduce some of the negative consequences of hearing loss. User-controllability promotes empowerment, positively influencing self-management, self-efficacy and participation (58).

Generally, users were satisfied with the self-administered hearing and cognitive tests, but conversely if they were not noticing improvement or it lacked usefulness there was a negative effect. This can lead to disengagement with the technology, in keeping with previous research (59). Importantly, non-use of offered interventions may not be a neutral experience as might be assumed, but rather a negative one for the individual. The potentially negative effects of offering such technology mean that e-health interventions in audiology cannot be adopted uncritically and require careful evaluation of negative as well as positive impact.

Likewise, it was apparent that instruction and feedback of the testing protocol and results needed to be clear and simple in order for the user to understand them and derive meaning. A previous study showed that patients not only experience negative emotions with abnormal results but even with normal results. Simply supplying access to information is counter-productive and guidance is needed on the interpretation (60). The variety of responses to the Auditory Training test suggests that its usefulness depends on the user's hearing loss and ability to listen in noise. This would support the idea of providing auditory training in a less universal manner, by tailoring it to the patient's hearing needs and their ability and willingness to use the app.

The speech-in-babble test is closer to real world listening situations than the pure-tone-audiogram (61). Whilst this test was designed as a tool to monitor improvement in performance, a number of users found that it helped promote awareness of real hearing challenges day-to-day, which allowed the user to “acclimatise” to the listening situation. It also allowed a demonstration of difficulties to significant others. This shows that this type of test can be used diagnostically but possibly more importantly it is also a powerful tool to aid rehabilitation and support the 4 P's model.

Users did not utilise the 4 kHz audiometry monitoring function very often. The main reason for this according to the patient comments was because it did not work as well as intended. The initial calibration often failed due to insufficient room conditions e.g., high ambient sound level or it crashed. It was not compulsory to complete this test routinely, only if the patient was exposed to an uncomfortably loud sound. A number of users commented here that they “didn't use” or “had not had a reason” to use this test. In relation to this there were very few comments relating to noise exposure even among younger users despite the fact that the app alerts the user when the hearing aids detect loud noise via a notification, and they are advised to perform an audiometry test. The questionnaire did not directly ask about this so it is limited how much we can infer from this. It could be an indication over a lack of awareness within the population about the level of noise that can have a detrimental impact on hearing health. The findings of current research available about awareness of excessive noise levels and noise-induced hearing loss (NIHL) is mixed (62–68). All of these studies primarily sampled younger adults. Those that showed most awareness were students, and there were differences between countries and communities. To our knowledge there are no studies that look at NIHL awareness in the typical older hearing aid population. Our comments or lack of in light of these studies hints at the need to actively educate and lead patients on noise exposure. This task of not only raising awareness on the impact of loud noise but also on protection could easily be implemented by hearing healthcare professionals.

A limitation of the study is that it was conducted, by necessity in accordance with ethical practice, on a group of participants who had agreed to use the technology and were aware of this when they signed up to the study. This could mean that the results were obtained on a particularly well-motivated and suited group of participants. However, to combat this limitation the large numbers of participants sampled involved goes some way to obviate this effect and therefore improve the generalisability of the results, since we expect that a range of participants would be included from within this limitation.

This study was part of a much bigger project designed to improve hearing care at the public health level and so the suite of tests and interventions had purposes other than direct patient care, meaning that the feedback data described in this study was collected as a cross sectional study without a control group and the EVOTION intervention was not intended as the optimum for patient care. However the results as presented are of interest both in relation to clinical care, public health and also the design of any similar future studies aimed at collecting big data sets for hearing health at the population level.

In conclusion, this study showed that 68% of participants were satisfied or very satisfied with overall use of the EVOTION system. Participants rated highly the experience of controlling hearing aids using a smartphone app. Ease of use is a critical factor in satisfaction and design emphasis must be placed on minimising technical issues such as poor connectivity between devices. Negative consequences of poor functionality must also be borne in mind. The majority of users, including the older adults who are the typical demographic within a hearing loss clinic, can utilise eHealth and are willing to adopt a mobile app paired with a wearable to complement their rehabilitation and health care. The older participants reported significantly greater satisfaction with connectivity and there were no significant differences between age, or gender, for all other items of the app including overall satisfaction of use. Although overall satisfaction was high across all groups, there were distinct differences in levels of satisfaction between different participating countries, illustrating that acceptability of technological interventions may vary in different populations. However, if the technology works well, is tailored to the individual and in-depth personalised guidance and support is provided, interventions such as the EVOTION system could be a key tool in maximising hearing aid uptake, promotion of self-management and improving outcomes for the growing health issue of hearing loss.

## Data Availability Statement

The raw data supporting the conclusions of this article will be made available by the authors, without undue reservation on reasonable request.

## Ethics Statement

The studies involving human participants were reviewed and approved by London Southeast Research Ethics Committee, NHS Health Research Authority. The patients/participants provided their written informed consent to participate in this study.

## Author Contributions

LM, MS, HW, D-EB, AB, DKi, AO, and NP collected data and contributed to review of the manuscript. HW and MS designed the feedback data collection, collated and analysed the data, and wrote the first draft of the manuscript. LM collated inputs, analysed data, and revised the manuscript. IK and DKo designed and created the app. All authors contributed to the article and approved the submitted version.

## Funding

This project has received funding from the European Union's Horizon 2020 research and innovation programme under grant agreement No. 727521.

## Acknowledgements

We would like to thank the participants who contributed to the study, and all the EVOTION partner organisations (list available at www.h2020evotion.eu).

## Conflict of Interest

The authors declare that the research was conducted in the absence of any commercial or financial relationships that could be construed as a potential conflict of interest.

## Publisher's Note

All claims expressed in this article are solely those of the authors and do not necessarily represent those of their affiliated organizations, or those of the publisher, the editors and the reviewers. Any product that may be evaluated in this article, or claim that may be made by its manufacturer, is not guaranteed or endorsed by the publisher.
